# Mitochondrial dysfunction in Parkinson's disease and aging

**DOI:** 10.3389/fnagi.2026.1914316

**Published:** 2026-10-28

**Authors:** Xiaoyu Lu, Zhiyan Huang, Zhongming Ying

**Affiliations:** 1 Department of Neurology, Taizhou Hospital of Chinese Traditional and Western Medicine, Taizhou, Zhejiang, China; 2 The First Affiliated Hospital of Zhejiang Chinese Medical University (Zhejiang Provincial hospital of Chinese medicine), Hangzhou, Zhejiang, China

**Keywords:** aging, calcium homeostasis, mitochondria, mitochondrial dynamics, oxidative phosphorylation, Parkinson's disease

## Abstract

Parkinson's disease (PD) is a neurodegenerative disorder for which advancing age is the major risk factor. Aging and PD share several features, including chronic neuroinflammation and pathological protein accumulation. This overlap suggests that mechanisms driving the aging process may also contribute to the onset and progression of PD. In this narrative review, we examine mitochondrial dysfunction as a shared feature of aging and PD, focusing on energy production, calcium buffering, apoptosis, inflammation, and reactive oxygen species (ROS) generation. The literature reviewed here suggests shared alterations in mitochondrial dynamics, increased mitochondrial DNA (mtDNA) instability, NAD^+^ depletion, Complex I inhibition, redox imbalance, and disrupted calcium homeostasis. We also summarize mitochondria-targeted interventions investigated in PD.

## 1 Introduction

Parkinson's disease (PD) is a neurodegenerative disorder characterized by the progressive degeneration of dopaminergic neurons in the substantia nigra of the midbrain and the formation of Lewy bodies, which consist primarily of misfolded a-synuclein (a-Syn). These are hallmark pathological features of the disease (Bloem et al., 2021; Hayes, 2019). Clinical manifestations include motor symptoms, such as bradykinesia and resting tremor, as well as non-motor symptoms, including hyposmia and cognitive impairment. To date, no effective treatment can halt or reverse PD-related neurodegeneration. Current management relies mainly on dopaminergic medications, including levodopa and dopamine agonists, which provide temporary relief of motor symptoms (Marsili et al., 2025). Aging is recognized as the most prominent non-genetic risk factor for PD (Coleman and Martin, 2022; Vijiaratnam et al., 2021). In a community-based study across six Latin American countries, PD prevalence increased from 1.0% among participants aged 65–69 years to 3.5% among those aged 80 years or older. These estimates illustrate the association between age and PD prevalence within the surveyed populations (Collier et al., 2017; Ma et al., 2025). The two conditions also share pathological features at the cellular and molecular levels. Cellular senescence and the senescence-associated secretory phenotype (SASP) are among these shared features. Senescent cells accumulate in brain tissue and release pro-inflammatory factors, chemokines, and proteases collectively referred to as the SASP, thereby promoting chronic low-grade inflammation. This disrupts the neuronal microenvironment and accelerates neurodegeneration (Rawji et al., 2016). Similar changes have been observed in PD, in which glial cells, including astrocytes and microglia, become activated and exhibit senescence-like phenotypes. SASP factors released by these cells, such as interleukin-6 (IL-6), may further impair neuronal function (Xia et al., 2020). At the molecular level, impaired proteostasis represents another important shared mechanism, primarily involving dysfunction of the autophagy-lysosomal pathway and the ubiquitin-proteasome system. In PD, mutations in several disease-associated genes, including GBA1, LRRK2, and ATP13A2, can impair lysosomal function. This compromises the clearance of toxic protein aggregates, such as misfolded a-Syn, leading to their accumulation within neurons and the formation of Lewy bodies (Ma et al., 2025; Sidransky et al., 2009; Dehay et al., 2012; Bonello et al., 2019; Jahan et al., 2026). Lewy-body-like aggregates have also been observed in the aging brain (Vernier et al., 2004). Failure of protein-clearance systems may therefore represent a shared basis for neuronal vulnerability in aging and PD. This brief comparison of aging and PD reveals substantial mechanistic overlap. Given the dependence of neurons on mitochondrial function, this review examines mitochondrial mechanisms linking aging and PD and discusses their implications for PD pathogenesis and treatment.

## 2 Mitochondrial dysfunction as a key contributor to aging and Parkinson's disease

Because of their complex cellular architecture and functional demands, neurons rely heavily on mitochondria to support a wide range of cellular processes and are therefore particularly vulnerable to mitochondrial dysfunction (Vaiasicca et al., 2024). A single neuron may contain thousands of mitochondria, making the maintenance of mitochondrial health essential (Jahan et al., 2026). Mitochondrial dysfunction is now considered one of the hallmarks of aging. On the one hand, mitochondria in senescent cells may become enlarged and elongated, while fission can be enhanced under certain senescence-associated conditions, resulting in the accumulation of damaged mitochondria. The accumulated dysfunctional organelles further increase ROS production and may impair the clearance of damaged mitochondria, ultimately aggravating cellular decline. On the other hand, disruption of mitochondrial function and integrity is also an important contributor to PD progression. Studies have reported marked impairment of the electron transport chain (ETC) in PD, accompanied by mitochondrial swelling, abnormal mitophagy, ROS accumulation, and enhanced inflammatory responses. Understanding the specific roles of mitochondria in aging and PD requires consideration of several coordinated processes, including mitochondrial dynamics, mtDNA maintenance, energy metabolism, oxidative regulation, and calcium homeostasis. The following sections examine these pathways and their alterations in aging and PD (Figure 1).

**Figure 1:**
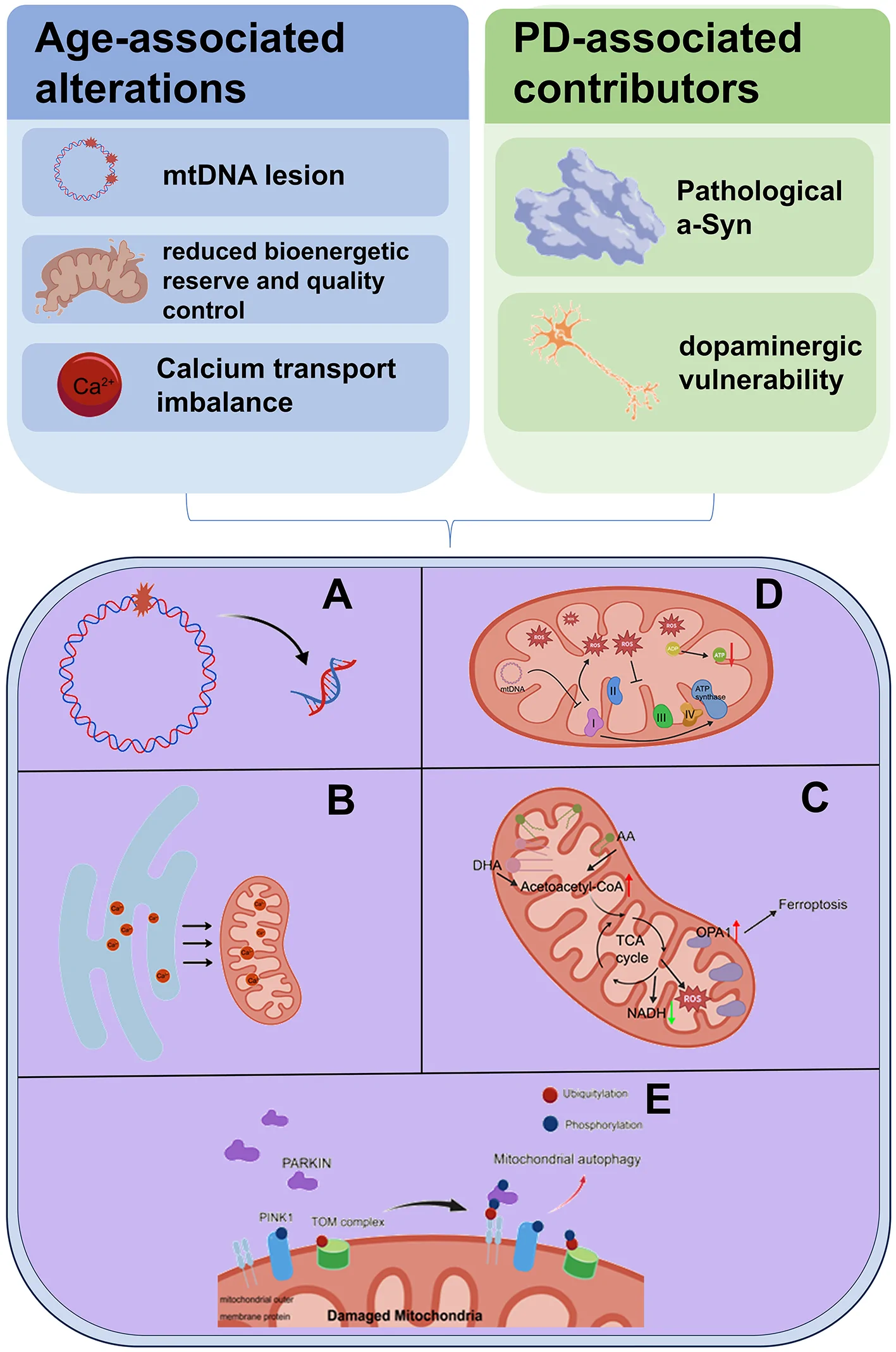
Common features of mitochondrial dysfunction in aging and Parkinson's disease (PD): **(A)** mitochondrial DNA damage; **(B)** calcium ion imbalance; **(C)** disruption of the tricarboxylic Acid Cycle; **(D)** respiratory chain dysfunction; and **(E)** impaired PINK1/PARKIN-mediated mitophagy. Created by BioGDP.

### 2.1 Mitochondrial dynamics and mitophagy

Mitochondrial quality and quantity are essential for maintaining cellular homeostasis, integrity, and function. To preserve mitochondrial homeostasis, damaged organelles must be selectively degraded through a process known as mitophagy (Picca et al., 2023; Lu et al., 2023). Mitophagy plays an important role in development, stress responses, aging, and programmed cell death.

#### 2.1.1 PINK1-mediated mitophagy

The PINK1/PARKIN-dependent ubiquitin-mediated pathway is currently the most studied mitophagy pathway. PINK1 phosphorylates ubiquitin and the ubiquitin-like domain of PARKIN, thereby promoting PARKIN recruitment to damaged mitochondria and relieving its autoinhibition to facilitate activation (Wauer et al., 2015). Activated PARKIN ubiquitinates outer mitochondrial membrane proteins. This ubiquitination can promote the proteasomal degradation of selected membrane proteins and generate signals that facilitate autophagic clearance of damaged mitochondria (Harper et al., 2018). PARKIN-mediated ubiquitination of outer mitochondrial membrane proteins marks damaged mitochondria for recognition by autophagy receptors, including OPTN and NDP52. These receptors facilitate the sequestration of damaged mitochondria within autophagosomes, which subsequently fuse with lysosomes to enable degradation of the mitochondrial cargo (Mary et al., 2023; Nguyen et al., 2016). Age-associated impairment of mitochondrial quality control has been reported in experimental models, although the contribution of PINK1/PARKIN-dependent mitophagy may vary across tissues and experimental conditions. Studies suggest that activation of PINK1/PARKIN-mediated mitophagy can facilitate the removal of dysfunctional mitochondria, reduce tissue ROS levels, and inhibit apoptosis, thus helping preserve cellular structure and function (Zhao et al., 2024). In PD, dysfunction of PINK1/PARKIN-mediated mitophagy is considered an important pathogenic mechanism. Defects in this pathway can arise from mutations in the relevant genes; mutations in either PINK1 or PRKN can cause autosomal recessive early-onset PD. Other PD-associated mutations may also disrupt this pathway. LRRK2 mutations have been reported to reduce mitophagic flux and weaken the interaction between PARKIN and outer mitochondrial membrane proteins (Xiong and Yu, 2024). Heterozygous GBA1 mutations can impair the recruitment of PARKIN, NBR1, and LC3 to damaged mitochondria, thereby reducing mitophagic flux (Smith and Schapira, 2022; Milenkovic et al., 2022).

Dysregulation of this pathway undermines mitochondrial quality control, resulting in the accumulation of damaged mitochondria, cellular energy deficiency, disturbed calcium homeostasis, and increased ROS, which can ultimately lead to neuronal dysfunction and death. This suggests that PINK1/PARKIN-mediated quality control is an important molecular link between aging and the pathogenesis of PD.

PINK1 and PARKIN also have non-canonical immunomodulatory functions (Matheoud et al., 2016; Ramirez et al., 2022). Under physiological conditions, PINK1 and PARKIN suppress antigen presentation through the mitochondrial-derived vesicle (MDV) pathway. When their function is impaired, inflammatory conditions may activate this pathway, engage immune cells such as microglia, and potentially contribute to immune-mediated neuronal injury (Rasmussen et al., 2026). Thus, loss of PINK1/PARKIN function may cause not only a cell-autonomous mitochondrial metabolic dysfunction but also inflammation-associated neuronal injury by relieving PINK1/PARKIN-mediated suppression of immune-related vesicular trafficking.

#### 2.1.2 Other pathways related to mitophagy and mitochondrial quality control

Mitochondria-associated membranes (MAMs) are another important component of mitochondrial quality control. Studies have reported increased oxidation of chaperone proteins at MAMs in senescent cells, leading to structural changes and ultimately impairing endoplasmic reticulum (ER) function (Silva-Palacios et al., 2025). Effective ER-mitochondria contacts are important for mitochondrial fission, fusion, and lipid metabolism. In a mouse study, mitochondria switched between pyruvate and fatty acids as fuel sources depending on feeding status (Randle, et al.). In young mice, phosphorylation of mitochondrial PDH-E1a increased during the daytime, suggesting reduced mitochondrial pyruvate oxidation during fasting (Nagayach et al., 2025). Mitochondria also undergo structural changes in response to nutrient availability (Liesa and Shirihai, 2013). In addition, OPA1 abundance in mouse mitochondria has been reported to vary diurnally, with higher levels during the day (Gong et al., 2015). OPA1 promotes the formation of oxidative phosphorylation (OXPHOS) complexes and supercomplexes, thereby increasing ATP production. During aging, disruption of these mitochondrial circadian rhythms may contribute to oxidative stress and the accumulation of damaged mitochondria. Overall, these findings suggest that mitochondria have important protective and regulatory roles during age-related cellular decline. In addition to removing damaged mitochondria to maintain mitochondrial homeostasis, accumulating evidence indicates that mitochondria and mtDNA can move within neuron-glia networks (Miliotis et al., 2019). In a rat model of PD, intranasally delivered mitochondria were observed to enter dopaminergic neurons in the substantia nigra that project to the striatum via anterograde transport and to reach dopaminergic neurons in the contralateral striatum through interhemispheric connections. This was associated with improved mitochondrial function, neuronal survival, and behavioral outcomes (Chang et al., 2021). In cellular models, PD-associated CHCHD2 mutations disrupt its interaction with CHCHD10 and compromise the integrity of the MICOS complex, which maintains mitochondrial cristae, ultimately causing mitochondrial dysfunction (Zhou et al., 2019). Related findings have also been reported in mouse and Drosophila models (Meng et al., 2017; Fan et al., 2023).

### 2.2 Loss of mitochondrial DNA stability

Mutations accumulate in mtDNA during aging. Because mtDNA encodes essential OXPHOS subunits, these mutations may compromise mitochondrial respiratory function. The proportion of mutated mtDNA within the total mtDNA pool can increase, resulting in greater heteroplasmy. DNA polymerase γ (POLG) is a key enzyme responsible for mtDNA replication. In mouse models, POLG deficiency can increase mtDNA replication errors, alter mtDNA abundance, and lead to premature aging (Trifunovic et al., 2004; Kujoth et al., 2005; Perez et al., 2023). In addition to replication-associated mutations, mtDNA can be damaged by exposure to ROS. Age-associated mitochondrial dysfunction increases ROS production, which can directly cause mtDNA base damage and strand breaks, leading to point mutations and deletions (Monzio Compagnoni et al., 2020). The efficiency of pathways that maintain mtDNA integrity, including base-excision repair, also declines with age, impairing timely repair and further accelerating mutation accumulation (Gredilla et al., 2010; Santos et al., 2013). For example, mutations in DRP1 can increase mitochondrial fission, potentially promoting the age-dependent accumulation of damaged mitochondria. The accumulation of damaged mitochondria, together with disruption or mutation of fusion proteins such as MFN1 and MFN2, can impair mitochondrial fusion (Kim et al., 2019). In brain tissue from patients with PD, substantial accumulation of mtDNA deletions has been observed, particularly in the substantia nigra-striatal system, at levels exceeding those in age-matched controls. This suggests that mtDNA deletions may contribute to PD pathogenesis (Borsche et al., 2021). mtDNA instability may therefore provide a mechanistic link between aging and PD onset.

### 2.3 Mitochondrial metabolic dysfunction

#### 2.3.1 Defects in the electron transport chain

Mitochondria generate cellular energy through OXPHOS, a process in which the ETC establishes a proton gradient that drives ATP synthesis. During aging, dysfunction of Complex I (CI) is a central contributor to declining ETC performance. Several core CI subunits are encoded by mtDNA, and age-associated mutations or deletions have been reported to alter CI structure and function in mouse models (Xu et al., 2025). A bioinformatics study also identified widespread age-related abnormalities in CI phosphorylation and damage to its core regions, potentially impairing electron transfer and proton pumping (Gowthami et al., 2019; Stefanatos and Sanz, 2011; Qi et al., 2023). Electron leakage increases superoxide and other ROS, whereas a weakened proton gradient reduces ATP production. Together, these processes create a vicious cycle between impaired mitochondrial energy metabolism and oxidative stress. Notably, studies in cells and mice suggest that CI dysfunction reduces NAD^+^ levels, disrupts the NAD^+^/NADH ratio, and decreases SIRT3 activity, thereby accelerating cellular senescence (Yao et al., 2024; Wang et al., 2022; Wiley et al., 2016). Restoration of the NAD^+^/SIRT3 axis has been reported to alleviate stress-induced senescence phenotypes caused by high glucose (Li et al., 2024). Reduced mitochondrial complex activity has also been observed in patients with PD. In particular, CI activity is markedly decreased in mitochondria from the substantia nigra and striatum, and its subunits show oxidative damage, functional impairment, and defective assembly (Choong and Mochizuki, 2023; Qadri et al., 2021). Environmental neurotoxins, including MPTP and related compounds, can selectively inhibit CI and induce persistent parkinsonian symptoms (Niwa and Maegawa, 2021; Ekstrand and Galter, 2009; Li et al., 2024; Innos and Hickey, 2021; Ibarra-Gutiérrez et al., 2023). However, these observations alone do not establish whether CI deficiency initiates disease or accompanies progressive PD (González-Rodŕıguez et al., 2021). Further experiments have shown that disrupting CI in mouse dopaminergic neurons produces progressive symptoms resembling human PD, supporting an important role for CI dysfunction in pathogenesis. mtDNA damage may also contribute to PD by compromising ETC complex integrity. In the neuron-specific TFAM-knockout MitoPark model, mtDNA dysfunction in dopaminergic neurons leads to ETC complex defects and a characteristic parkinsonian phenotype (Harrington et al., 2023). These findings indicate that CI impairment can contribute to the emergence and progression of PD-like features in animal models and may be a key link between aging and PD. Collectively, CI dysfunction increases ROS and reduces ATP, further damaging mtDNA and mitochondrial proteins; mtDNA damage in turn worsens ETC dysfunction, including CI impairment, thereby promoting both aging and PD progression.

#### 2.3.2 Dysregulation of the tricarboxylic acid cycle

Dysfunction of the tricarboxylic acid (TCA) cycle causes abnormal substrate metabolism and is a key component of mitochondrial dysfunction (Harrington et al., 2023). During aging, the activities of key TCA-cycle enzymes decline. Inhibition of the a-ketoglutarate dehydrogenase complex (a-KGDHC), for example, induces senescence in human Hs68 fibroblasts and reduces cycle efficiency, potentially causing incomplete oxidation or accumulation of metabolic substrates such as acetyl-CoA and decreasing NADH production (Vaiasicca et al., 2024). TCA-cycle dysfunction has also been observed in the substantia nigra of patients with PD. Excess a-ketoglutarate (a-KG) derived from glutamine metabolism via glutaminase 2 (GLS2) can enter the TCA cycle and promote excessive ROS generation by the ETC (Le et al., 2012; Hu et al., 2010; Gao et al., 2015). Notably, under PD-associated stress, the intracellular distribution of acetyl-CoA becomes pathologically imbalanced, with reduced nuclear acetyl-CoA and increased mitochondrial acetyl-CoA (Ding et al., 2026). Reduced nuclear acetyl-CoA decreases histone H3K27 acetylation at the OPA1 promoter and suppresses OPA1 transcription. Meanwhile, mitochondrial acetyl-CoA accumulation promotes acetylation of OPA1 at the inner mitochondrial membrane and reduces its activity. Impaired OPA1 function triggers mitochondrial fragmentation, iron accumulation, lipid peroxidation, ROS generation, and reduced OXPHOS, ultimately promoting mitochondria-associated ferroptosis (Liao et al., 2023; Liang et al., 2024). The SIRT3/ACSS2/OPA1 axis has been implicated in regulating acetyl-CoA reprogramming to resist ferroptosis. SIRT3 deacetylates and activates acetyl-CoA synthetase 2 (ACSS2), facilitating redistribution of acetyl-CoA from mitochondria to the nucleus. This promotes OPA1 transcription and deacetylation, maintains mitochondrial homeostasis, suppresses ferroptosis, and protects dopaminergic neurons during PD progression (Samant et al., 2014; Ding et al., 2026). In vivo studies have reported that pharmacological activation of SIRT3 or AAV-mediated OPA1 overexpression alleviates ferroptosis, protects dopaminergic neurons, and improves motor function in PD mouse models (Ding et al., 2026). In addition, a-Syn mutations or overexpression can activate ATP-citrate lyase (ACLY), disrupting acetyl-CoA homeostasis and the function of the histone acetyltransferase p300 in human neurons. ACLY is a key enzyme in acetyl-CoA production, and its inhibition has been reported to rescue pathological phenotypes in PD neuronal, organoid, zebrafish, and mouse models (Son et al., 2025). Thus, age-related decline in TCA-cycle efficiency may create a metabolically vulnerable state that facilitates PD onset, while PD pathology further aggravates metabolic imbalance, jointly contributing to neuronal energy failure and death. Mitochondria are also the principal site of fatty-acid B-oxidation (Houten et al., 2016), and numerous studies have reported abnormalities in mitochondrial lipid metabolism during aging (Byrns et al., 2024). An energy crisis can trigger compensatory activation of mitochondrial fatty-acid oxidation, producing excess acetyl-CoA that may upregulate senescence-associated proteins and accelerate senescence. The mitochondrial enzyme ACSS1, which converts acetate to acetyl-CoA, is functionally regulated by acetylation. Genetically engineered mice mimicking sustained ACSS1 acetylation (Acss1K635Q/K635Q) exhibit metabolic disturbances and increased hepatocyte senescence markers, including p21 and senescence-associated B-galactosidase (SA-B-gal) (Xu et al., 2025). In PD, lipid accumulation arising from abnormal mitochondrial lipid metabolism is considered one of the key drivers of ETC defects. Intracellular accumulation of long-chain polyunsaturated fatty acids, such as arachidonic acid (AA) and docosahexaenoic acid (DHA), can reduce mitochondrial biogenesis and impair ETC function, leading to excessive ROS accumulation. Abnormal overproduction of acyl-CoA caused by lipid metabolic disturbances can also disrupt TCA-cycle homeostasis and reduce electron transfer to CI. CI dysfunction leads to NADH accumulation, which further disrupts the TCA cycle through feedback inhibition (Bogie et al., 2020; Saiki et al., 2017; Yang et al., 2025).

### 2.4 Disruption of calcium homeostasis

Abnormal Ca^2+^ signaling is an indicator of cellular decline during aging (Petrova et al., 2024). The ER is a central organelle for regulating Ca^2+^ homeostasis. As adjacent organelles, mitochondria and the ER form close structural contacts known as mitochondria-associated membranes (MAMs). MAMs are important for transferring Ca^2+^ from the ER to mitochondria and can thereby promote mitochondria-dependent apoptosis (Rizzuto et al., 1998; Dematteis et al., 2025). Several proteins at MAMs regulate the extent and dynamics of Ca^2+^ transfer. Among them, the inositol 1,4,5-trisphosphate receptor (IP_3_R) is a Ca^2+^ channel on the ER membrane; binding of IP_3_ to IP_3_R mediates Ca^2+^ release from the ER into the cytoplasm. Disrupted calcium homeostasis is considered one factor contributing to the selective vulnerability of dopaminergic neurons. Studies suggest that the ER-associated protein TRPC3, which regulates IP_3_ receptors, is downregulated with age, altering Ca^2+^ transfer from the ER to mitochondria. This is accompanied by increases in mitochondrial oxygen-consumption rate (OCR) and overall OXPHOS, ultimately triggering a ROS burst and NAD^+^ depletion, inducing p53-dependent cell-cycle arrest, and promoting the SASP, thereby accelerating cellular senescence (Farfariello et al., 2022). In models of neurodegenerative disease, Ca^2+^ transfer between the ER and mitochondria is more complex. Several studies have found that Parkin participates in the formation of ER-mitochondria contact sites. Parkin overexpression in HeLa and SH-SY5Y cells increases ER-mitochondria contacts and is associated with increased mitochondrial Ca^2+^ uptake and ATP production (Basso et al., 2018). Conversely, ER-mitochondria connectivity is increased in Parkin-deficient cells and mice (Gautier et al., 2016). In Drosophila models, parkin mutations increase ER stress, and reducing ER-mitochondria contacts under conditions of mitochondrial injury may be neuroprotective (Celardo et al., 2016). These findings suggest that the quality, rather than simply the number, of ER-mitochondria contact sites may determine their effects and may help explain differences among PD models. Pathological proteins in PD, including mutant a-Syn, have also been reported to reduce calcium transfer (Erustes et al., 2021; Paillusson et al., 2017). Calcium dysregulation should therefore not be viewed simply as an increase or decrease in calcium levels. Age-related decline in calcium-regulatory capacity may create conditions favorable to PD onset, while PD-associated mitochondrial dysfunction may render neurons more susceptible to calcium dysregulation, together promoting neurodegeneration.

## 3 Mitochondria-associated mechanisms contributing to progressive neuronal loss

### 3.1 DJ-1 protein

DJ-1 is an important oxidative-stress sensor and antioxidant factor and has also been described as a downstream effector in PINK1/PARKIN-mediated mitophagy. DJ-1 is generally located in the cytoplasm but can translocate to mitochondria and the nucleus under oxidative stress (Irrcher et al., 2010). In PD, structural abnormalities and loss of DJ-1 function are associated with the pathogenesis of autosomal recessive early-onset PD. DJ-1 deficiency or mutation increases ROS generation and reduces ATP production. The role of DJ-1 in mitophagy remains controversial: under some experimental conditions, both increased DJ-1 expression and its inhibition appear to benefit model animals, and the reasons remain unclear. In PD animal models, inhibition of DJ-1-induced autophagy can impair a-Syn clearance and exacerbate toxic aggregation, whereas DJ-1 overexpression can reduce neuronal injury by inducing autophagy (Xu et al., 2017). p62 is also a component of pathogenic inclusions observed in PD. Confocal imaging has shown that DJ-1 overexpression reduces the number of p62-positive areas in midbrain dopaminergic neurons in mice, consistent with an increase in autophagy (Gao et al., 2012). In response to MPP^+^ neurotoxicity, oxidized DJ-1 increases significantly in the SN4741 mouse dopaminergic cell line and acts as a mitophagy substrate, mediating mitochondrial clearance that protects cells from MPP^+^-induced death. Increased oxidized DJ-1 has also been observed in brain tissue from patients with PD (Wang et al., 2016). Based on these findings, we hypothesize that age-related reductions in DJ-1 may suppress mitophagy and contribute to PD-related pathology. Further investigation is required to clarify the precise role of DJ-1.

### 3.2 The vicious cycle between a-syn and mitochondria

Abnormal accumulation of a-Syn, encoded by SNCA, is a central pathological mechanism in PD, and similar changes have also been observed during aging. Studies of rat brain tissue have found that a-Syn can interact directly with and enter mitochondria, where it affects mitochondrial metabolism, particularly through CI (Martínez et al., 2018). A study in non-human primates identified abnormal phosphorylation of a-Syn at serine 129 as a key feature of pathological aggregation in both PD and aging. This process is accompanied by increased total a-Syn levels and Lewy-body formation. In PD, pathological a-Syn aggregates, particularly preformed fibrils (PFFs), can spread between neurons; their uptake and intercellular propagation contribute to disease progression in the brain (Han et al., 2024). Mutant a-Syn can also disrupt tethering complexes, including VAPB-PTPIP51 and IP_3_R-GRP75-VDAC, reducing ER-mitochondria contacts and calcium transfer (Paillusson et al., 2017; Erustes et al., 2021). On the one hand, pathological a-Syn aggregates interact with mitochondria through their N-terminal targeting sequence, inhibit CI activity, disrupt calcium transfer, and directly impair mitochondrial function. Mutant a-Syn can also impair mitochondrial function and inhibit autophagy through pathways such as ACLY, further compromising its own clearance (Rousseaux et al., 2016). On the other hand, mitochondrial dysfunction and oxidative stress promote abnormal a-Syn modification and aggregation. These reciprocal effects jointly contribute to dopaminergic neurodegeneration (Kiechle et al., 2019). A study of postmortem PD tissue found that a-Syn binds to the outer mitochondrial membrane translocase TOM20, inhibiting the interaction between TOM20 and TOM22 and ultimately impairing mitochondrial function and protein import (Di Maio et al., 2016). Some studies have proposed that physiological a-Syn can adopt helical tetrameric assemblies in equilibrium with monomeric forms. Under in vitro conditions, helical a-Syn oligomers are resistant to aggregation (Bartels et al., 2011), whereas unfolded monomers readily aggregate into insoluble, B-sheet-rich amyloid fibrils (Conway et al., 2000). An in vitro mitochondrial study suggests that altered a-Syn proteostasis in the PD context may destabilize physiological a-helical oligomers, leading to accumulation of aggregation-prone monomers and formation of prefibrillar oligomers with mitochondrial toxicity. These oligomers contribute to CI-dependent dysfunction and ultimately to degeneration of vulnerable neuronal populations (Luth et al., 2014). Thus, pathological a-Syn aggregation in the context of aging and PD may be an important contributor to mitochondrial dysfunction.

### 3.3 Ferroptosis

Ferroptosis is an iron-dependent form of regulated cell death driven by the accumulation of phospholipid peroxides. Mitochondrial glutathione accounts for approximately 10-15% of total cellular glutathione, and mitochondria lack the enzymes required to synthesize glutathione; it must therefore be imported from the cytoplasm (James et al., 2009). The transporter SLC25A39 is an important mediator of glutathione import into mitochondria, and suppression of SLC25A39 expression reduces mitochondrial glutathione levels (Wang et al., 2021). Gialluisi et al. identified SLC25A39 as a candidate gene associated with PD and reported its co-expression with tyrosine hydroxylase in human dopaminergic neurons of the substantia nigra (Shi et al., 2024). During aging, regulation of the mitochondrial labile iron pool (mLIP) declines, resulting in iron accumulation (Ding et al., 2026). Excess iron catalyzes ROS generation through the Fenton reaction and triggers lipid peroxidation of polyunsaturated fatty acids (PUFAs) in mitochondrial membranes. This damages membrane integrity and depletes glutathione (GSH), further weakening antioxidant defenses (Tábara et al., 2025; Dai et al., 2023). In PD models, expression of SLC25A28, a protein involved in mitochondrial iron uptake, is markedly increased (Carroll et al., 2011). Iron deposition has also been observed in the substantia nigra of patients with PD (Yang et al., 2016). Excess ROS and iron overload act synergistically to exacerbate lipid peroxidation and directly induce mitochondria-dependent ferroptosis (Gao et al., 2022). Protective proteins such as mitochondrial ferritin (FtMt) may also become dysfunctional in PD, impairing iron sequestration and suppression of the Fenton reaction and thereby weakening protection against ferroptosis (Guan et al., 2017). Although iron deposition has been observed in mitochondria in both aging and PD, most evidence comes from experimental models; it remains uncertain whether iron accumulation is a causal driver or merely an accompanying phenomenon.

### 3.4 Chronic neuroinflammation

During aging, the body enters a state of chronic low-grade inflammation known as inflammaging, which provides a background for neuroinflammation. Accumulation of senescent cells is a key driver of this process. Senescent cells, including astrocytes and microglia in the brain, secrete pro-inflammatory factors, chemokines, and proteases—a phenomenon known as the SASP—that continuously disrupts the neuronal microenvironment (Franceschi et al., 2018; Xilouri et al., 2013). Mitochondrial dysfunction can promote inflammation through the release or mislocalization of mitochondrial DNA (mtDNA). Depending on its subcellular localization and the cellular context, mtDNA can engage distinct inflammatory pathways. Endolysosomal mtDNA can stimulate TLR9 signaling, whereas cytosolic oxidized mtDNA has been implicated in NLRP3 inflammasome activation. In experimental PD models, microglial oxidized mtDNA has been shown to interact with NLRP3 and promote caspase-1 activation and IL-1B release. These findings support multiple links between mitochondrial damage and neuroinflammation, rather than a single obligatory TLR9–NLRP3 signaling sequence (Bernardini et al., 2019; Naren et al., 2023; Zhou et al., 2022). In addition, misfolded and aggregated a-Syn, a hallmark pathological protein in PD, can itself act as a DAMP and directly activate microglia to release inflammatory mediators, including IL-1B, IL-6, and tumor necrosis factor-a (TNF-a). These mediators can directly injure neurons and further compromise blood-brain barrier integrity, creating a self-sustaining vicious cycle (Cribbs et al., 2012; Mattson and Arumugam, 2018). Inflammatory mediators released by activated glial cells exacerbate mitochondrial dysfunction and oxidative stress, while damaged mitochondria generate additional damage-associated molecular pattern (DAMPs) that feed back into inflammatory pathways. Chronic inflammation can also impair autophagy–lysosomal clearance, promoting the accumulation of pathological proteins such as a-Syn (Mou et al., 2022; Herrick and Tansey, 2021; Collins et al., 2012). Neuroinflammation is therefore both a consequence of aging and PD and a potential driver of ongoing neurodegeneration (Figure 2).

**Figure 2:**
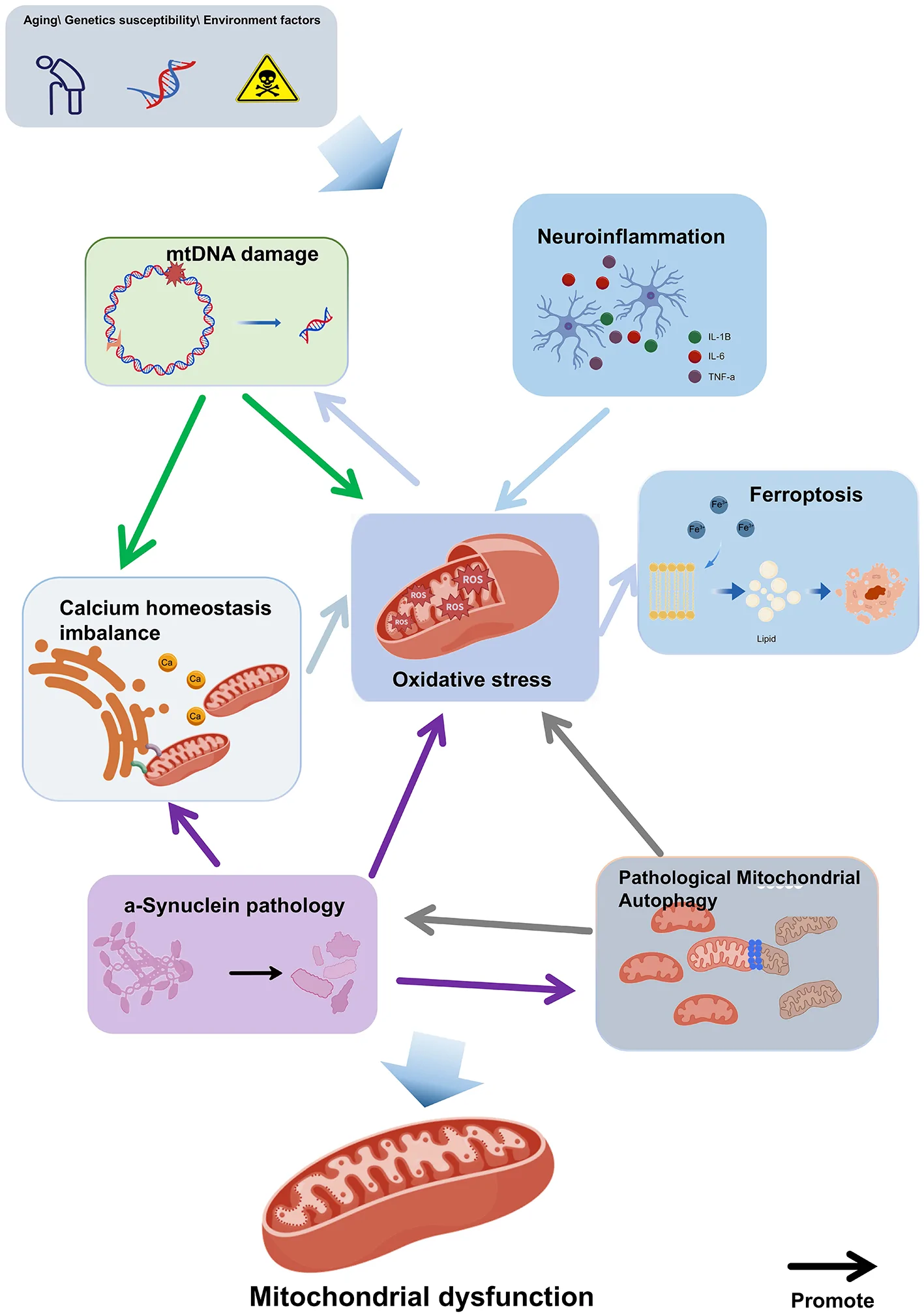
Mitochondrial mechanisms contributing to dopaminergic neurodegeneration in Parkinson's disease. Created by BioGDP.

## 4 Therapeutic strategies targeting mitochondrial dysfunction in PD: pre-clinical and clinical evidence

Studies of compounds that ameliorate mitochondrial dysfunction suggest potential therapeutic applications in PD. In animal models, mitochondria-targeted antioxidants, including MitoQ, MitoVitE, and MitoTEMPOL, reduce mitochondrial oxidative stress and improve motor function (Langley et al., 2017; Liu et al., 2023; Prasuhn et al., 2021). N-acetylcysteine (NAC), a precursor of glutathione, has been reported to improve symptoms and dopaminergic neuronal function in a rat model when administered subcutaneously (Rahimmi et al., 2015). Strategies that maintain cellular energy homeostasis through metabolic regulation have also shown potential. Glucagon-like peptide-1 (GLP-1) receptor agonists, such as exenatide, have been reported to protect dopaminergic neurons against MPTP toxicity, increase dopamine levels, and improve motor performance in MPTP-treated diabetic rats (Harkavyi et al., 2008; Li et al., 2009; Elbassuoni and Ahmed, 2019). A randomized, double-blind phase II clinical trial investigated liraglutide in patients with PD (NCT02953665). The study included 37 participants in the active-treatment group and 18 in the placebo group. Among patients receiving standard PD treatment, subcutaneous liraglutide administered for 52 weeks was associated with significant improvement in activities of daily living (Malatt et al., 2022). NAD^+^ precursors, such as nicotinamide riboside (NR), showed improvement in selected metabolic biomarkers in patients with newly diagnosed PD in the phase I NADPARK study (Schöndorf et al., 2018). Research on mitophagy has found that spermidine can induce mitophagy and mitochondrial biogenesis in aged mice (Messerer et al., 2023). Kaempferol and paeonol glycosides have been reported to improve the survival and function of glutamatergic and cholinergic neurons and improve memory in mouse models (Xie et al., 2022). Urolithin A and tomatidine have been shown to extend the lifespan of Caenorhabditis elegans by stimulating mitophagy (Ryu et al., 2016; Brakedal et al., 2022) (Table 1).

**Table 1:** Mitochondria-targeted therapeutic strategies for Parkinson's disease.

Pathological process	Research stage and evidence type	Therapeutic agent	Proposed mechanism	Model or population
Oxidative stress	Clinical RCT	MitoQ	Mitochondria-targeted antioxidant activity	Patients with PD
Oxidative stress	Pre-clinical mechanistic evidence	MitoVitE	Mitochondria-targeted antioxidant activity	Experimental oxidative-stress models
Oxidative stress	Pre-clinical mechanistic evidence	MitoTEMPOL	Modulation of mitochondrial ROS	Experimental oxidative-stress models
Oxidative stress	Pre-clinical PD-model evidence	N-acetylcysteine (NAC)	Support of glutathione-related antioxidant defense	Rotenone-treated rats
Metabolic dysfunction	Clinical RCT	Exenatide	GLP-1 receptor signaling; proposed metabolic support	Patients with PD
Metabolic dysfunction	Exploratory clinical evidence (secondary-outcome signal)	Liraglutide	GLP-1 receptor signaling; proposed metabolic support	Patients with PD
Metabolic dysfunction	Phase I safety and biomarker evidence; clinical efficacy unestablished	Nicotinamide riboside (NR)	NAD^+^ replenishment	Newly diagnosed patients with PD
Impaired mitochondrial quality control	Pre-clinical aging-model evidence	Urolithin A	Promotion of mitophagy-related quality control	*C. elegans*
Impaired mitochondrial quality control	Pre-clinical aging-model evidence	Tomatidine	Promotion of mitophagy-related quality control	*C. elegans*

PD, Parkinson's disease; RCT, randomized controlled trial; GLP-1, glucagon-like peptide-1; ROS, reactive oxygen species.

## 5 Conclusions and future prospects

The pathogenesis of PD is complex, and no curative treatment is currently available. This review identifies multiple pathological features shared by aging and PD, including impaired mitochondrial quality control, mitochondrial metabolic dysfunction, and disrupted calcium homeostasis, which may inform our understanding of PD-related pathological changes. Studies have shown that experimentally induced defects in mitochondrial morphology and function can reproduce irreversible PD-like features in animal models, suggesting that mitochondrial injury may contribute to disease pathogenesis. However, most animal models of PD and aging do not fully reproduce the neurochemical changes observed in humans, which limits mechanistic interpretation. In addition, many studies of therapeutic efficacy remain at the pre-clinical stage and require further investigation. Although diverse strategies targeting mitochondria are being explored for PD, translation from pre-clinical models to clinical practice remains difficult and time-consuming. The effects of interventions targeting mitophagy and mitochondrial metabolism may depend on disease stage, cell type, and the extent of pathway modulation: manipulating mitophagy or energy metabolism may be protective under some conditions but may cause further mitochondrial injury under others. Consequently, modulation of a single target may carry a risk of adverse effects.
